# It's not just the television: survey analysis of sedentary behaviour in New Zealand young people

**DOI:** 10.1186/1479-5868-8-132

**Published:** 2011-12-01

**Authors:** Louise S Foley, Ralph Maddison, Yannan Jiang, Timothy Olds, Kate Ridley

**Affiliations:** 1 Clinical Trials Research Unit, University of Auckland, Private Bag 92019, Auckland Mail Centre, Auckland 1142, New Zealand; 2 Health and Use of Time (HUT) Group, Sansom Institute for Health Research, University of South Australia, GPO Box 2471, Adelaide, South Australia 5001, Australia; 3 School of Education, Flinders University, GPO Box 2100, Adelaide, South Australia 5001, Australia

**Keywords:** Sedentary behaviour, self-report, accelerometry, cross-sectional survey

## Abstract

**Background:**

Sedentary behaviour has been linked with adverse health outcomes in young people; however, the nature and context of being sedentary is poorly understood. Accurate quantification and description of sedentary behaviour using population-level data is required. The aim of this research was to describe sedentary behaviour among New Zealand (NZ) youth and examine whether sedentary behaviour differs by Body Mass Index (BMI) status in this population.

**Methods:**

A national representative cross-sectional survey of young people aged 5-24 years (n = 2,503) was conducted in 2008-2009. Data from this survey, which included subjectively (recall diary; n = 1,309) and objectively (accelerometry; n = 960) measured sedentary behaviour for participants aged 10-18 years were analysed using survey weighted methods.

**Results:**

Participants self-reported spending on average 521 minutes per day (standard error [SE] 5.29) in total sedentary behaviour, 181 minutes per day (SE 3.91) in screen-based sedentary activities (e.g., television and video games), and 340 minutes per day (SE 5.22) in other non-screen sedentary behaviours (e.g., school, passive transport and self-care). Accelerometer-measured total sedentary behaviour was on average 420 minutes per day (SE 4.26), or 53% (SE 0.42%) of monitored time. There were no statistically significant differences in time spent in sedentary behaviour among overweight, obese and healthy/underweight young people.

**Conclusions:**

Both subjective and objective methods indicate that NZ youth spend much of their waking time being sedentary. No relationships were found between sedentary behaviour and BMI status. These findings extend previous research by describing engagement in specific sedentary activities, as well as quantifying the behaviour using an objective method. Differences in what aspects of sedentary behaviour the two methods are capturing are discussed. This research highlights the potential for future interventions to target specific sedentary behaviours or demographic groups.

## Background

Sedentary behaviour in young people has been linked with adverse health outcomes including increased metabolic risk [1] and adiposity [2,3]. It is proposed that changes in the physical and social environment encourage sedentariness across the full spectrum of behavioural contexts, including work, school, home and transport [4]. Decreasing sedentary time has emerged as an important target for health promotion in conjunction with efforts to promote increased participation in physical activity [5]. However, how and where people are being sedentary is poorly understood, with sedentary behaviour research to date focussing predominantly on leisure-time screen-based pursuits such as television viewing [6]. Accurate quantification and description of sedentary behaviour using population-level data is vital to understand this phenomenon and inform the development of public health interventions.

Sedentary behaviour is not merely the absence of physical activity; rather, it involves purposeful engagement in a large variety of behaviours associated with low energy expenditure [7]. Derived from the Latin sedere ("to sit"), sedentary behaviour encompasses both non-leisure and leisure activities including school work, socialising, passive transport, screen time and non-screen leisure activities such as arts and crafts and musical pursuits [8]. Sedentary behaviour has been measured both subjectively and objectively [4]. Subjective (self-report) measures have the advantage of providing detail on the type and context of sedentary behaviour, but are associated with the inherent limitations of all self-report instruments such as social desirability and recall biases [9]. Early research studies involving young people tended to quantify sedentary behaviour using self-reported screen time as a proxy for total sedentary time [6]. However, recent research has utilised use-of-time self-report tools to capture a wider variety of sedentary behaviours [8,10]. For example, a national survey of Australian young people aged 9-16 years (n = 2,200) using a use-of-time tool indicated that non-screen sedentary behaviours constituted 60% of total sedentary time. Moreover, screen time was only moderately correlated with total sedentary time (r = 0.53), indicating that screen time may not be appropriate as a proxy for total sedentary time [8].

Sedentary behaviour has also been quantified objectively using accelerometry. Objective measures overcome the biases associated with self-report. They are also associated with low participant burden; however, information regarding the context or nature of the behaviour is not obtained. There is also considerable debate about how sedentary behaviour should be operationalised when using objective measurement devices. Accelerometry has recently been used to quantify sedentary behaviour in several large population-based studies. In a representative cross-sectional survey conducted in the United States (US), Americans aged 6-85 years spent 460 (standard error [SE] 2.4) minutes per day in sedentary behaviour [5]. Adolescents (16-19 years) were one of the most sedentary groups, spending 482 (SE 4.8) minutes per day in sedentary pursuits [5]. A longitudinal study of American adolescent females indicated that sedentary behaviour increased from 461 (standard deviation [SD] 67) to 512 (SD 64) minutes per day between the ages of 11-13 years [11]. Similarly, a United Kingdom (UK)-based longitudinal sample found 11 year old children engaged in 428 (SD 66.4) minutes of sedentary behaviour per day [3]. Because of the relative advantages and disadvantages of subjective and objective measurement of sedentary behaviour, a dual approach using both methods concurrently may be optimal to identify both the types and overall amount of sedentary behaviour young people engage in. Together, this approach has the potential to improve our understanding of when, where and how young people are sedentary.

The overall objective of this research was to comprehensively describe sedentary behaviour among New Zealand (NZ) young people aged 10-18 years using a dual subjective and objective approach to measurement. Specific aims were to a) quantify self-reported engagement in behavioural sets of sedentary behaviours, broadly classified as screen and non-screen behaviours; b) objectively quantify total sedentary time using accelerometry; c) examine sedentary behaviour outcomes by various demographic sub-groups and d) examine whether sedentary behaviour differs between overweight, obese, and healthy (or underweight) young people. To our knowledge, this is the first study to report concurrently-collected subjective and objective data on sedentary behaviour in a nationally representative sample of children, though this has been done recently in adults [12].

## Method

A national representative cross-sectional survey of NZ young people aged 5-24 years was conducted between September 2008 and May 2009. The survey was conducted according to the ethical principles outlined in the Declaration of Helsinki and was covered by Statistics New Zealand Tier 1 ethical approval. Written consent was obtained from all participants or their parent, depending on the age of the participant. Sedentary behaviour outcomes for participants aged 10-18 years are reported here.

### Study population and design

A complex survey design involving stratified multi-stage sampling was used, with meshblocks (a defined geographic area) as the primary sampling unit. Within each meshblock, eligible households were identified and asked to participate. One young person was randomly chosen from each eligible household. The overall response rate was 55%. A total of 2,503 young people aged 5-24 years participated in the survey, which consisted of 18.8% Māori (indigenous population), 9.6% Pacific, 12.9% Asian and 71.4% NZ European. This is representative of the ethnic composition of the general NZ population [13]. A total of 1,315 participants were aged 10-18 years.

### Procedure

Data were collected during a face-to-face home visit and a subsequent telephone interview conducted 7-14 days after the home visit. During the home visit, height and weight were measured and data on demographics and self-reported (subjective) physical activity and sedentary behaviour were collected. Accelerometers were then fitted to participants to provide an objective measure of behaviour over a seven-day period. During the telephone interview, additional self-reported data on physical activity and sedentary behaviour were collected.

### Measures

Height was measured to the nearest 0.1 cm with a stadiometer (Seca, 214, Hamburg, Germany) and weight was measured to the nearest 0.1 kg with a digital scale (Tanita, UM-070, Illinois, US) according to standard procedures [14]. For both height and weight, two measures were taken. A third measurement was performed if differences of 0.1 cm and 0.1 kg, respectively were observed between the first and second measurements. The mean of two or the median of three measurements was used in analysis. BMI was calculated from the weight (kg) divided by height (m) squared. International Obesity Task Force [15] classifications of body size were derived.

Self-reported sedentary behaviour was measured using the Multimedia Activity Recall for Children and Adolescents (MARCA) [16]. The MARCA is a computerised use-of-time tool. All daily activities (including sleep) are retrospectively recalled in sequential time segments of five minutes or more for the previous 24 hours. Participants choose from a list of approximately 250 activities. Each activity is linked to an energy cost taken from existing compendia in children [17] and adults [18]. Metabolic Equivalents (METs) [19] are used describe the intensity of activities. The MARCA has been shown to have adequate psychometric properties [16,20]. For the current survey up to four days of recall were completed. Participants recalled the two previous days of activity at each of the two data collection periods.

Sedentary behaviour was measured objectively using an Actigraph accelerometer (model AM7164-2.2C). Accelerometers have been validated as an objective measure of physical activity in children [21], adolescents and young adults [22,23], and have also been used to quantify sedentary behaviour [3,5,11]. All participants were asked to wear the Actigraph during waking hours for seven consecutive days (including two weekend days). A ten-second epoch was used [21], and data were summed to provide minute-by-minute measurement.

### Data treatment

#### MARCA

MARCA-derived total sedentary time (TST) was defined as waking seated or lying activities at < 3 METs, as listed in the MARCA compendium. The majority of included activities fell in the range of 2 METs or less; of the 70 activities classified as sedentary, only seven were associated with an energy expenditure of greater than 2 METs.

Screen sedentary time (SST) was defined as the number of waking minutes reported in seated or lying activities at < 3 METs involving television (sitting or lying), video games, computers or movies and was comprised of five activities. Watching a movie at the cinema was classified under the "movie" category, but watching a movie on the television was classified as "television". Non-screen sedentary time (NSST) was defined as the number of waking minutes reported in seated or lying activities at < 3 METs not involving a screen, and comprised the remaining 65 activities. NSST was further broken down into six intuitive sets: socialising, school/work, self-care, passive transport, music and leisure.

TST, SST and NSST derived from available data were divided by the number of days to provide a daily average for each participant. In the total survey population aged 5-24 years, 2,493 (99.6%) participants provided valid MARCA data, among whom 1,309 (52.5%) were aged 10-18 years.

#### Accelerometer

Accelerometer-derived TST was defined as number of minutes spent in activity eliciting between zero and 100 accelerometer counts per minute [24]. Using published criteria [25] a valid minute was defined as a recorded minute that did not fall into a sequence of ≥ 20 minutes of zero activity counts. A valid day was defined as a recorded day that had a minimum of 600 valid minutes. Average daily TST was calculated from valid days using valid minutes only. To account for potential differences in valid minutes of accelerometer data between participants, TST was also expressed as a percentage of valid minutes recorded for each participant. In the total survey population aged 5-24 years, 1,812 (72.4%) participants provided valid accelerometer data, among whom 960 (53.0%) were aged 10-18 years.

### Statistical analysis

Statistical analyses were performed using statistical software SAS version 9.2 (SAS Institute Inc, Cary NC) and R version 2.12.0 (R Foundations for Statistical Computing). Survey weights appropriate for stratified multistage samplings were calculated to estimate the population means and SEs using the jack-knife replication method. For all variables, data are presented descriptively. Demographic sub-groups were defined including gender, age, deprivation, area (rural vs. urban), ethnicity and overweight status. Deprivation level was defined according to the 2006 NZ Deprivation Index [26] (I = least deprived, V = most deprived). Ethnicity was evaluated using total response which showed the counts of all responses given for each ethnic group [27,28]. International Obesity Task Force criteria were used to define overweight status from BMI [15,29].

Weighted regression analysis was conducted to examine whether time spent in various sedentary behaviours differed among overweight, obese, and healthy/underweight children (i.e. BMI status), adjusting for their age in years, gender, prioritised ethnicity, and NZ Deprivation Index. A total of 1,300 participants aged 10-18 years who provided valid BMI data were included in this analysis. MARCA derived TST, SST and NSST, as well as accelerometer-derived TST, were considered as the outcomes of interest. BMI status was used as the independent predictor (underweight and healthy weight were combined due to very small numbers).

## Results

### MARCA

**Figure 1:**
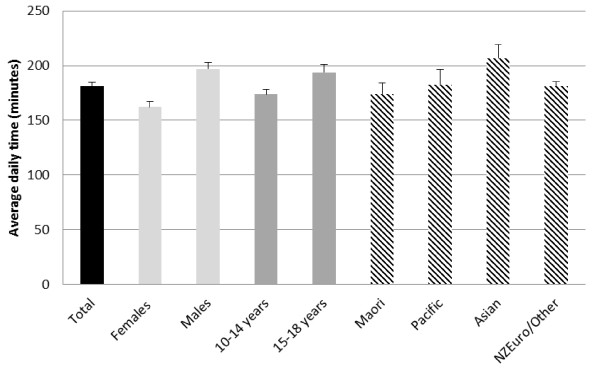
**Self-reported (MARCA) screen sedentary time by population sub-group**.

**Figure 2:**
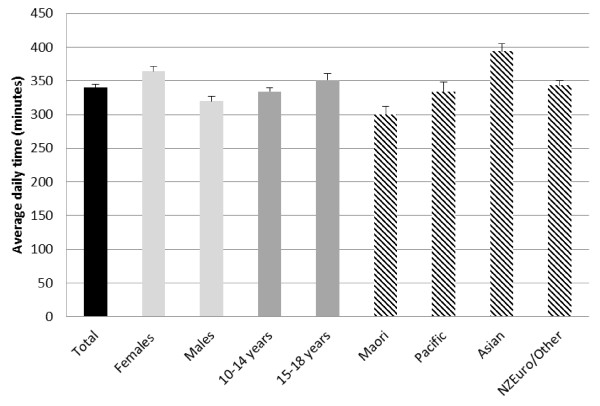
**Self-reported (MARCA) non-screen sedentary time by population sub-group**.

Daily time (minutes) spent in TST, SST and NSST for participants aged 10-18 years is presented in Table 1. Overall, young people reported engaging in 521 minutes (SE 5.29) of sedentary behaviour per day, comprised of 181 (SE 3.91) minutes of screen-based activities (Figure 1) and 340 (SE 5.22) minutes of non-screen activities (Figure 2).

**Table 1:** Daily engagement in sedentary behaviour types derived by self-report (MARCA)

Variable	Total sedentary time (min)			Screen sedentary time (min)			Non-screen sedentary time (min)		
	*n^1^*	*Mean^2^*	*SE^3^*	*n^1^*	*Mean^2^*	*SE^3^*	*n^1^*	*Mean^2^*	*SE^3^*

All	1,309	521.1	5.29	1,309	181.1	3.91	1,309	340.0	5.22

Gender									

female	606	526.4	7.64	606	162.3	5.01	606	364.1	7.62

male	703	516.6	7.30	703	196.9	5.78	703	319.7	6.75

Age group									

10-14 years	825	508.0	6.43	825	174.0	4.45	825	334.0	5.68

15-18 years	484	544.4	9.14	484	193.6	7.63	484	350.8	9.69

Deprivation									

I	275	541.9	13.89	275	168.5	6.29	275	373.4	13.05

II	264	528.6	9.50	264	191.4	9.90	264	337.2	8.69

III	277	528.1	11.35	277	179.1	8.11	277	349.0	10.91

IV	216	518.7	10.35	216	178.0	9.63	216	340.7	11.22

V	272	491.8	12.54	272	188.5	9.75	272	303.3	12.57

Area									

rural	209	520.1	10.81	209	177.3	9.17	209	342.8	10.16

urban	1,100	521.2	5.80	1,100	181.5	4.26	1,100	339.7	5.74

Ethnicity									

Māori	248	474.1	12.55	248	174.1	10.15	248	300.0	12.14

Pacific	131	516.4	13.39	131	182.5	13.80	131	333.9	13.79

Asian	174	600.5	10.30	174	206.8	12.62	174	393.7	11.23

NZ Euro/Other	923	524.0	6.50	923	181.0	4.48	923	343.0	6.73

OW status									

underweight	61	532.7	26.88	61	201.1	16.16	61	331.6	24.53

healthy	788	523.2	6.62	788	179.4	5.57	788	343.9	6.47

overweight	303	522.7	9.99	303	176.6	7.81	303	346.1	10.87

obese	147	495.8	15.85	147	189.7	11.03	147	306.1	14.60

^1^Number of participants in each category

^2^Weighted mean estimate

^3^Standard error of the mean

MARCA = Multimedia Activity Recall for Children and Adolescents

For SST, males reported higher use of sedentary screen technologies than females (197 minutes per day [SE 5.78] versus 162 minutes per day [SE 5.01], respectively). Older participants reported greater SST than younger participants (194 minutes per day [SE 7.63] in 15-18 year olds versus 174 minutes per day [SE 4.45] in 10-14 year olds). Asian participants reported higher use of screen-based technologies (207 minutes per day [SE 12.62]) than any other ethnic group. Daily time (minutes) spent in categories of SST is presented in Table 2. Television watching was the predominant screen-based activity, with reported use of 132 minutes per day (SE 3.25) in the whole sample.

**Table 2:** Daily engagement in categories of screen sedentary time derived by self-report (MARCA)

Variable	Television (min)			Computer/video game (min)			Movie (min)		
	*n^1^*	*Mean^2^*	*SE^3^*	*n^1^*	*Mean^2^*	*SE^3^*	*n^1^*	*Mean^2^*	*SE^3^*

All	1,309	131.9	3.25	1,309	46.7	2.32	1,309	2.5	0.46

Gender									

female	606	126.2	4.30	606	33.0	2.67	606	3.1	0.78

male	703	136.8	4.81	703	58.3	3.51	703	1.9	0.54

Age group									

10-14 years	825	131.8	3.83	825	40.4	2.48	825	1.8	0.51

15-18 years	484	132.2	5.88	484	57.8	4.56	484	3.6	0.90

Deprivation									

I	275	116.1	5.09	275	48.4	4.78	275	3.9	1.48

II	264	134.6	8.99	264	53.1	5.32	264	3.7	1.29

III	277	129.0	5.96	277	47.6	4.20	277	2.4	0.96

IV	216	127.6	7.40	216	49.2	7.02	216	1.2	0.59

V	272	151.1	8.28	272	36.1	4.40	272	1.2	0.51

Area									

rural	209	137.4	6.94	209	38.6	5.07	209	1.3	0.77

urban	1,100	131.2	3.56	1,100	47.7	2.53	1,100	2.6	0.51

Ethnicity									

Māori	248	139.1	8.51	248	34.4	4.30	248	0.7	0.30

Pacific	131	149.8	12.76	131	29.5	4.41	131	3.1	1.50

Asian	174	132.0	9.77	174	72.6	7.92	174	2.3	1.28

NZ Euro/Other	923	131.1	3.75	923	47.2	2.77	923	2.8	0.58

OW status									

underweight	61	136.4	11.95	61	61.5	13.70	61	3.1	2.00

healthy	788	125.9	4.51	788	50.6	3.19	788	2.9	0.67

overweight	303	135.7	6.65	303	39.5	4.28	303	1.4	0.47

obese	147	153.1	11.25	147	34.4	4.32	147	2.3	1.40

^1^Number of survey participants in each category

^2^Weighted estimate of the mean

^3^Standard error of the estimate calculated using the replicated weights

MARCA - Multimedia Activity Recall for Children and Adolescents

For NSST, females reported higher engagement in non-screen based sedentary activities than males (364 minutes per day [SE 7.62] versus 320 minutes per day [SE 6.75] respectively). Those of the lowest deprivation level reported the greatest amount of NSST (373 minutes per day [SE 13.05]), compared with those in the highest deprivation level who reported the least (303 minutes per day [SE 12.57]). Asian participants reported more NSST (394 minutes per day [SE 11.23]) than any other ethnic group. Daily time (minutes) spent in categories of NSST is presented in Table 3. Overall, school/work activities were the most frequently reported (145 minutes per day; SE 4.55), followed by socialising (45 minutes per day; SE 2.07), passive transport (42 minutes per day; SE 1.47), sedentary leisure activities (39 minutes per day; SE 2.01) and music (11 minutes per day; SE 0.97).

**Table 3:** Daily engagement in categories of non-screen sedentary time derived by self-report (MARCA)

Variable	Socialising (min)			School/work (min)			Passive transport (min)			Music (min)			Leisure (min)		
	*n^1^*	*Mean^2^*	*SE^3^*	*n^1^*	*Mean^2^*	*SE^3^*	*n^1^*	*Mean^2^*	*SE^3^*	*n^1^*	*Mean^2^*	*SE^3^*	*n^1^*	*Mean^2^*	*SE^3^*

All	1,309	45.1	2.07	1,309	145.2	4.55	1,309	42.4	1.47	1,309	10.8	0.97	1,309	38.8	2.01

Gender															

female	606	55.3	2.90	606	147.5	7.10	606	45.2	2.32	606	11.9	1.29	606	40.9	2.76

male	703	36.5	2.32	703	143.3	6.06	703	40.0	1.71	703	9.9	1.38	703	37.1	2.57

Age group															

10-14 years	825	36.1	2.03	825	153.5	4.34	825	38.3	1.41	825	9.6	0.87	825	41.5	2.32

15-18 years	484	61.1	3.78	484	130.5	9.75	484	49.7	3.06	484	12.9	2.19	484	34.1	3.25

Deprivation															

I	275	45.0	3.38	275	160.6	9.66	275	51.1	2.95	275	10.1	1.57	275	48.3	4.43

II	264	37.0	3.33	264	146.3	7.56	264	45.5	2.79	264	8.9	1.61	264	41.3	4.50

III	277	42.0	4.27	277	156.8	11.86	277	41.8	2.72	277	10.8	1.67	277	38.5	3.16

IV	216	56.1	6.99	216	128.3	10.73	216	40.3	4.92	216	14.2	2.59	216	39.8	6.40

V	272	45.0	4.18	272	135.1	11.09	272	33.9	2.86	272	10.2	2.89	272	27.4	3.64

Area															

rural	209	40.5	3.73	209	144.2	8.83	209	56.5	3.08	209	9.6	2.17	209	37.8	3.93

urban	1,100	45.7	2.28	1,100	145.4	5.01	1,100	40.6	1.59	1,100	11.0	1.06	1,100	39.0	2.21

Ethnicity															

Māori	248	45.2	4.34	248	126.3	11.61	248	36.7	2.94	248	13.5	3.15	248	31.8	5.81

Pacific	131	57.4	6.85	131	139.4	12.24	131	38.2	3.17	131	11.1	2.13	131	37.4	6.16

Asian	174	41.4	4.36	174	188.7	10.41	174	39.4	2.75	174	8.3	1.40	174	43.3	4.59

NZ Euro/Other	923	44.4	2.55	923	141.1	5.61	923	45.2	1.86	923	10.2	0.92	923	41.8	2.30

OW status															

underweight	61	39.3	8.93	61	134.9	18.81	61	36.2	5.41	61	6.7	2.90	61	47.7	12.43

healthy	788	45.8	2.82	788	142.7	5.47	788	43.9	1.95	788	12.2	1.43	788	38.1	2.20

overweight	303	45.8	3.83	303	155.9	12.66	303	42.9	2.53	303	8.6	1.55	303	44.6	4.59

obese	147	42.9	5.09	147	135.2	10.17	147	35.2	4.01	147	9.6	1.69	147	28.7	3.91

^1^Number of survey participants in each category

^2^Weighted estimate of the mean

^3^Standard error of the estimate calculated using the replicated weights

MARCA - Multimedia Activity Recall for Children and Adolescents

### Accelerometer

**Figure 3:**
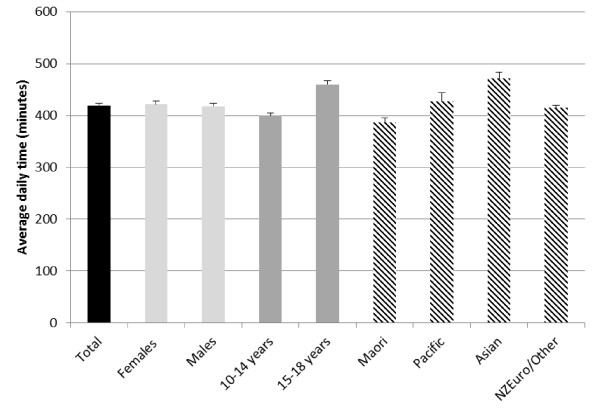
**Objectively measured (accelerometer) total sedentary time by population sub-group**.

Daily time (minutes) spent in TST is presented in Table 4 and Figure 3. Young people aged 10-18 years spent 420 minutes per day (SE 4.26) engaged in sedentary behaviour, or 53% (SE 0.42%) of monitored time. TST was similar for females and males. However, there were differences between age groups; the younger age group (10-14 years) spent 400 minutes per day (SE 4.95) in sedentary behaviour (50% [SE 0.48%] of monitored time), compared with 460 minutes per day (SE 7.64) (58% [SE 0.74%] of monitored time) in the older age group (15-18 years). Participants of Asian ethnicity were the most sedentary of all ethnic groups, spending an average of 472 minutes per day (SE 11.49) engaged in sedentary behaviour (59% [SE 1.10%] of monitored time), compared with Māori who were the least sedentary, with an average of 386 minutes per day (SE 9.29) (49% [SE 0.88%] of monitored time).

**Table 4:** Daily engagement in sedentary behaviour derived by accelerometry

Variable	Daily valid accelerometer time (min)			Total sedentary time (min) (0 ≤ counts per minute ≤ 100)			Percentage of daily valid minutes spent in sedentary behaviour		
	*n^1^*	*Mean^2^*	*SE^3^*	*n^1^*	*Mean^2^*	*SE^3^*	*n^1^*	*Mean^2^*	*SE^3^*

All	960	789.6	4.71	960	419.5	4.26	960	52.9%	0.42%

Gender									

female	440	778.0	5.52	440	421.3	5.98	440	54.0%	0.63%

male	520	799.2	7.17	520	418.0	6.03	520	52.0%	0.57%

Age group									

10-14 years	646	789.5	5.70	646	399.8	4.95	646	50.4%	0.48%

15-18 years	314	789.7	7.54	314	459.7	7.64	314	58.1%	0.74%

Deprivation									

I	211	770.0	7.29	211	421.3	7.83	211	54.6%	0.91%

II	204	790.5	10.04	204	428.1	8.56	204	54.1%	0.84%

III	210	796.2	7.59	210	424.1	8.85	210	53.0%	0.87%

IV	152	787.5	10.18	152	402.6	12.13	152	50.7%	1.09%

V	180	805.6	15.01	180	418.9	11.11	180	51.7%	0.89%

Area									

rural	152	802.0	8.87	152	420.8	7.26	152	52.3%	0.88%

urban	808	787.9	5.20	808	419.3	4.73	808	53.0%	0.46%

Ethnicity									

Māori	166	789.2	10.27	166	386.1	9.29	166	48.6%	0.88%

Pacific	82	826.6	21.96	82	426.8	17.78	82	51.1%	1.42%

Asian	118	799.2	11.40	118	471.7	11.49	118	58.9%	1.10%

NZ Euro/Other	717	781.0	4.53	717	414.8	4.29	717	53.0%	0.46%

OW status									

underweight	52	756.9	18.32	52	424.0	15.04	52	56.0%	1.46%

healthy	591	790.4	5.62	591	423.4	5.63	591	53.3%	0.54%

overweight	215	788.2	8.50	215	412.9	7.42	215	52.3%	0.78%

obese	99	804.1	17.19	99	408.6	15.20	99	50.6%	1.26%

^1^Number of survey participants in each category

^2^Weighted estimate of the mean

^3^Standard error of the estimate calculated using the replicated weight

### Relationship between sedentary behaviour and BMI status

The adjusted survey regression analysis indicated no statistically significant differences between underweight/healthy weight, overweight and obese participants for time spent in sedentary behaviour.

For MARCA-derived SST, obese participants engaged in 14.5 minutes more per day than underweight/healthy weight participants (95% CI -13.4 to 42.4; p = 0.31). For MARCA-derived NSST, overweight participants engaged in 8.1 minutes more per day than underweight/healthy weight participants (95% CI -16.5 to 32.7; p = 0.52) and obese participants engaged in 15.0 minutes less per day than underweight/healthy weight participants (95% CI -46.3 to 16.3; p = 0.35).

## Discussion

The aim of this research was to describe sedentary behaviours among NZ young people aged 10-18 years. Results from both data sources indicate this group spend much of their waking time in sedentary pursuits, consistent with reports from other developed countries [3,5,30,31].

SST (most commonly television watching) has been reduced successfully in previous interventions, with concurrent improvements in weight indices [32]. SST is thought to have adverse effects on body composition via displacement of physical activity, reduction of metabolic rate, exposure to food advertising and promoting snacking [32], and adverse psychosocial effects through exposure to violence and adult content [33]. NZ young people used screen technologies for 181 (SE 3.91) minutes per day on average, somewhat lower than an Australian estimate of 230 (SD 114) minutes per day in adolescents aged 9-16 years in a survey that also used the MARCA [8]. However, these estimates are still markedly lower than recent data from the US, which found that 8-18 year olds reported spending 324 (no SD reported) minutes per day in SST as it is defined in this study [34].

Though SST is an important target for intervention, it should be noted that the daily time reported in NSST was nearly twice that of screen-based activities. NZ young people engaged in non-screen sedentary behaviour for 340 (SE 5.22) minutes per day on average. This estimate is very similar to a MARCA-derived NSST estimate of 345 (SD 105) minutes per day in Australian adolescents aged 9-16 [8]. Some non-screen activities such as school may be considered less discretionary and others such as reading may be considered more socially desirable than screen time. However, it is still possible to intervene to break up extended periods of sedentary time and increase energy expended. Modification of the school environment to allow standing lessons and opportunities for physical activity has been shown to significantly increase movement in children [35]. In adults, breaking up sedentary time has been associated with favourable body composition and metabolic risk, independent of total sedentary time and physical activity level [36]. It may also be appropriate to encourage displacement of sedentary socialising and transport with more active alternatives, as each accounted for approximately 45 minutes of daily activity in this sample.

The accelerometry data indicated that young people spent 420 minutes (SE 4.26) in sedentary behaviour per day. This estimate is broadly consistent with surveys from other developed countries conducted in adolescents using the 100 count per minute cut-point. Estimates for total sedentary time from other countries include 452 (SE 6.0) minutes per day in 12-15 year olds and 482 (SE 4.8) minutes per day in 16-19 year olds in a representative US sample [5], 512 (SD 54) minutes per day in a longitudinal US sample aged 13 years [11], 428 (SD 66.4) minutes per day in a longitudinal UK sample aged 11 years [3] and 496 (SD 80.6) and 471 (SD 84.3) minutes per day in a Spanish boys and girls, respectively, aged 13-16 years [30]. Consistent with the well-documented decline in physical activity from childhood through adolescence to adulthood [37], the accelerometry data also indicated an increase in sedentary behaviour with age. Children aged 10-14 years engaged in 60 minutes less daily sedentary behaviour than those aged 15-18 years (400 [SE 4.95] minutes or 50% of monitored time versus 460 [SE 7.64] minutes or 58% of monitored time, respectively). This indicates a disturbing trend in health-related behaviour across adolescence. However, contrary with reports from other countries [2,3], this study found no differences in engagement in total or specific types of sedentary behaviour between overweight and obese participants and their healthy or underweight counterparts.

The results of this study suggest that the most appropriate targets for intervening to reduce screen time in NZ are males, those in their late teens, and those of Asian ethnicity. Although television watching was the predominant screen behaviour, computer and video games were used for approximately 45 minutes per day. Moreover, interventions to reduce NSST in NZ young people may be more appropriate for females, who report greater levels of NSST than males, and those of Asian ethnicity who reported high levels of NSST as well as high levels of SST. For Asian participants, the MARCA data indicated a greater engagement in computer- and school-based sedentary behaviour. This likely reflects an academic rather than a leisure orientation in sedentary behaviours. Culturally-appropriate interventions should consider the nature of sedentary behaviour in this ethnic group.

The specific strengths of the study are the use of a large, representative sample of NZ young people and the use of the survey methodology for analysis. To the best of our knowledge, this is the first time that complementary methodologies (accelerometry and self-report) have been used to describe sedentary behaviour in a sample of children this large. In adults, it is recommended that population-based monitoring of sedentary time include both self-reported and device-based measurement [12]. However, several limitations warrant discussion. Firstly, this was a cross-sectional study, which does not allow for inferences of causality, or capture change across time. Secondly, there was a 45% non-participation rate and it is possible that non-participants differed from participants, though the study population was nationally representative by ethnicity, age and geography. During the original survey parents were approached to recruit their child, thus selection bias was based on parent rather than the young person, which might dilute this effect.

Data from a subset of participants aged 10-18 years were analysed rather than the total sample. Exclusion of those aged 19-24 years was considered the most appropriate approach to provide meaningful comparisons of sedentary behaviour amongst young people at a similar stage of development (pre-adolescent or adolescent), as this older age group are often at a transitional stage of life in which they move from school into sedentary, office-based occupations. Exclusion of those aged 5-9 years was for pragmatic reasons because of differences in the way data was collected. For this younger age group, a parent gave a proxy report of activities the child performed whilst being directly supervised by the parent. Therefore, all of the time spent at school was coded as "school", and no further information was provided about sedentary behaviour or physical activity during this time. This was not comparable with older participations who recalled a full 24 hours of activity. Finally, the use of a 10-18 year old age group allows for comparison with other international data sets.

The tools used to capture sedentary behaviour are each associated with their own limitations. As discussed, the MARCA data may be affected by the biases associated with self-report, though the psychometric properties have been shown to be sound, and there was a very high proportion of valid data. For accelerometry, it is important to note that no current consensus exists on the most appropriate way to define sedentary behaviour, with published definitions ranging from 100-1100 activity counts per minute (cpm) [38]. Furthermore, estimates relied on participant compliance with wearing the accelerometer during waking hours. When compared to the MARCA, a smaller proportion of participants provided valid data (99.6% vs. 72.4%).

Further discussion of issues associated with the measurement of sedentary behaviour via self-report and accelerometry is warranted. Sedentary behaviour is an emerging field of research in public health, but there remains little consensus regarding the optimal way of defining and measuring it. In this survey, the MARCA and accelerometry captured distinct aspects of sedentary behaviour. A seated or lying body posture was the key criterion for defining sedentary behaviour according to the MARCA, with a secondary MET threshold applied. For example, "watching TV sitting - 1.2 METs" was classified as a sedentary behaviour, whereas "playing the drums - 4.0 METs" was not, even though this was a seated behaviour. Conversely, accelerometry defines sedentary behaviour as low movement and does not provide any information on body posture. Standing still can elicit low accelerometer counts and therefore accelerometer-derived sedentary time will likely include a mixture of time spent both sitting and standing.

For total sedentary behaviour, estimates from the MARCA (521 [SE 5.29] minutes per day) were higher than those from accelerometry (420 [SE 4.26] minutes per day). The 100 minute discrepancy between methods raises the question as to which estimate best represents the "true" value. Differences in the operationalisation of sedentary behaviour, as well as differences in what the tools actually measure, most likely accounts for the discrepancy in outputs. Because of their objectivity, accelerometers have inherent appeal, but are not a criterion method for assessing sedentary behaviour. A new class of devices based on inclinometry (e.g., the activPAL) show promise for the objective assessment of body posture [39]. Though it relies on self-report, the MARCA has potential to provide additional information on what individuals do when they are sedentary. It is likely that the "true" value lies somewhere between the two estimates. Though the absolute value of the estimates differed between the two methods, the patterns of sedentary behaviour among groups (e.g., younger vs. older age groups, ethnic groups) were strikingly similar.

## Conclusions

In conclusion, data from both subjective and objective sources demonstrate that sedentary behaviour accounts for a significant proportion of waking time and occurs across the full spectrum of behavioural contexts in NZ young people. In total, young people spent approximately 420-520 minutes per day being sedentary, comprised of 35% screen activities and 65% non-screen activities. The dual approach to measurement used here provides complementary data on not only the volume of sedentary time, but the context of what people are doing. The results highlight the potential for tailoring a sedentary behaviour intervention to a demographic group of interest, based on the pattern and popular types of sedentary behaviour in that group. In particular, males and older adolescents may benefit from reducing sedentary screen time, whereas females may benefit more by reducing non-screen sedentary behaviours. Those of Asian ethnicity may consider reducing total sedentary time. This study highlights the importance of considering all sedentary behaviours when designing interventions.

## Competing interests

The authors declare that they have no competing interests.

## Authors' contributions

LF was responsible for the conception and design of the sedentary behaviour analyses and was primarily responsible for drafting the manuscript. RM was responsible for the design of the original national survey. YJ undertook the survey analyses. All authors (LF, RM, YJ, TO and KR) contributed to the interpretation of data, critical revision of the manuscript and have given final approval of the manuscript.
